# Evaluating the efficacy and safety of first-line immunotherapy for metastatic triple-negative breast cancer: a systematic review and network meta-analysis of randomized controlled trials with a focus on PD-L1 expression

**DOI:** 10.3389/fonc.2026.1797793

**Published:** 2026-06-05

**Authors:** Yanxiao Sun, Longtao Zhang, Dong Guo

**Affiliations:** 1 First Clinical Medical College, Shandong University of Traditional Chinese Medicine, Jinan, China; 2 College of Acupuncture and Tuina, Shandong University of Traditional Chinese Medicine, Jinan, China

**Keywords:** efficacy, immune checkpoint inhibitors, network meta-analysis, PD-L1, safety, triple-negative breast cancer

## Abstract

**Introduction:**

This study systematically reviewed randomized controlled trials (RCTs) and conducted a Bayesian network meta-analysis to evaluate first-line immunotherapy regimens for metastatic triple-negative breast cancer (mTNBC), aiming to compare the efficacy and safety of different immunotherapy combinations and to explore the impact of programmed cell death ligand 1 (PD-L1) expression levels on survival benefits in patients.

**Methods:**

A comprehensive literature search was conducted across PubMed, Embase, Cochrane Library, and Web of Science databases. Outcomes included overall survival (OS), progression-free survival (PFS), objective response rate (ORR), and the incidence of grade ≥3 adverse events (AE≥3). A Bayesian network meta-analysis was conducted to evaluate the efficacy and safety of different immunotherapy regimens in the overall population and various PD-L1 expression subgroups (≥1%, ≥10%, and <1%). This study has been registered on PROSPERO (ID: CRD420251138714).

**Results:**

A total of 8 RCTs involving 3,789 patients and 6 immunotherapy combination regimens were included. Compared with chemotherapy alone, the combination of immune checkpoint inhibitors (ICIs) with chemotherapy significantly improved OS (HR = 0.90, 95% CI: 0.82-0.98) and PFS (HR = 0.82, 95% CI: 0.75-0.89) in the overall mTNBC population. The AE≥3 was slightly higher (OR = 1.20, 95% CI: 0.94-1.53) without statistical significance. In patients with PD-L1 expression ≥1%, ICIs combined with chemotherapy significantly improved OS (HR = 0.83, 95% CI: 0.74-0.93) and PFS (HR = 0.74, 95% CI: 0.66-0.82). In patients with PD-L1 ≥10%, the benefits for OS (HR = 0.68, 95% CI: 0.53-0.87) and PFS (HR = 0.68, 95% CI: 0.52-0.91) were even more pronounced. Toripalimab combined with chemotherapy (Toripa-chemo) showed the greatest OS benefit in the overall population (HR = 0.58, 95% CI: 0.38-0.87). Atezolizumab combined with Entinostat and chemotherapy (Atezo-Entino-chemo) showed a trend toward improved OS (HR = 0.49, 95% CI: 0.22-1.12) and ORR (OR = 5.14, 95% CI: 0.70-37.94). Pembrolizumab combined with chemotherapy (Pembro-chemo) demonstrated the best safety profile (OR = 1.06, 95% CI: 0.52-2.16). Subgroup analysis showed that in patients with PD-L1 ≥ 1%, Toripa-chemo conferred the greatest benefit, with the most favorable OS (HR = 0.67, 95% CI: 0.40-1.12) and PFS (HR = 0.64, 95% CI: 0.47-0.87). Conversely, in patients with PD-L1 <1%, Pembro-chemo showed the best OS benefit (HR = 0.97, 95% CI: 0.72-1.31).

**Conclusions:**

Compared to chemotherapy alone, immunotherapy combined with chemotherapy significantly improves survival outcomes in mTNBC patients, with more pronounced benefits observed in the PD-L1 positive population. Toripa-chemo and Pembro-chemo demonstrate a balanced profile of efficacy and safety, suggesting that they may be suitable options for first-line treatment of mTNBC. Among these, Toripa-chemo may be considered a preferred first-line regimen for PD-L1 positive patients.

**Systematic review registration:**

https://www.crd.york.ac.uk/prospero/, identifier ID: CRD420251138714.

## 1 Introduction

Breast cancer (BC) is one of the most common malignancies worldwide. In 2022, approximately 2.31 million cases of breast cancer were diagnosed globally, accounting for 11.6% of all cancer cases and ranking second after lung cancer (1, 2). It is the most common cancer among women, representing approximately 25% of all newly diagnosed cases in females. In the same year, breast cancer resulted in approximately 666,000 deaths, accounting for 6.9% of all cancer-related deaths and ranking as the fourth leading cause of cancer mortality worldwide and the leading cause among women (1). BC can be classified into three main molecular subtypes based on the expression of molecular markers such as estrogen receptor (ER), progesterone receptor (PR), and human epidermal growth factor receptor 2 (HER2): hormone receptor (HR) positive, HER2 positive, and triple-negative breast cancer (TNBC) (3). TNBC accounts for approximately 15%-20% of all BC cases and has the highest rates of recurrence and mortality. More than 50% of TNBC patients experience recurrence within 3–5 years of diagnosis, making it the subtype with the highest recurrence and mortality rates (4, 5). Although breast cancer screening is becoming increasingly popular, the proportion of patients presenting with distant metastasis at diagnosis is significantly higher for TNBC than for other subtypes, primarily due to the aggressive nature of TNBC and the lack of effective early screening targets, contributing to the poor prognosis of this subtype (6).

Before the application of immune checkpoint inhibitors (ICIs) in clinical practice, first-line treatment for metastatic triple-negative breast cancer (mTNBC) mainly relied on chemotherapy. Common strategies included combination chemotherapy (such as albumin-bound paclitaxel combined with carboplatin) or single-agent sequential chemotherapy (such as taxanes, anthracyclines, gemcitabine, or capecitabine) (7). Although chemotherapy can provide clinical benefits for some patients, its efficacy is limited, with a median progression-free survival (PFS) of only about 5–6 months, and it is often associated with severe adverse events of grade 3 or higher, such as neutropenia and peripheral neuropathy, which significantly impact patient adherence to treatment and quality of life (8).

With the increasing use of ICIs, the treatment paradigm for mTNBC has undergone a significant transformation. Currently, multiple international guidelines recommend ICIs combined with chemotherapy as the new standard first-line treatment for eligible patients, offering a new therapeutic option for patients with mTNBC (9–11). ICIs work by blocking the programmed death protein 1/programmed cell death ligand 1 (PD-1/PD-L1) immune checkpoint pathway, releasing the tumor-induced suppression of T cells, and activating the body’s anti-tumor immune response, thereby improving patient survival (12). Studies have shown that ICIs and chemotherapy have a synergistic anti-tumor effect, where chemotherapy can induce immunogenic cell death in tumor cells, facilitate the release of tumor antigens, increase the expression of MHC class I molecules, and improve the tumor microenvironment, thereby enhancing the efficacy of ICIs (13). Based on the evidence from the KEYNOTE-355 phase III clinical trial, PD-1/PD-L1 inhibitors combined with chemotherapy have become the first-line standard treatment for PD-L1 positive mTNBC patients (8). PD-L1 expression is widely regarded as an important biomarker for predicting the efficacy of ICIs, with patients showing high PD-L1 expression typically experiencing more significant survival benefits from ICI treatment (10).

However, despite the availability of several high-quality randomized controlled trials, there is still a lack of direct head-to-head comparisons among different immune checkpoint inhibitor–based combination regimens. Moreover, substantial heterogeneity exists across studies in terms of treatment strategies and PD-L1 subgroup definitions, making it difficult to draw consistent conclusions regarding the optimal first-line regimen for mTNBC patients in clinical practice. In this context, a network meta-analysis provides a valuable approach to integrate both direct and indirect evidence, enabling a comprehensive comparison and ranking of multiple treatment options within a unified analytical framework. Therefore, this study aimed to perform a Bayesian network meta-analysis to systematically evaluate the efficacy and safety of different immunotherapy-based regimens, with particular emphasis on PD-L1-defined subgroups, in order to provide more robust and clinically relevant evidence for treatment decision-making.

## 2 Materials and methods

This network meta-analysis (NMA) was conducted in strict accordance with the Preferred Reporting Items for Systematic Reviews and Meta-Analyses extension statement for network meta-analyses (14) (**Supplementary Table 1**). Given the lack of head-to-head RCTs that directly compare different immunotherapy regimens, this study employed a Bayesian framework for indirect comparisons and to probabilistically assess the ranking of different treatment strategies in terms of efficacy and safety (15). To ensure transparency in the research process, reproducibility of results, and innovation in methods, the study protocol has been prospectively registered with the International Prospective Register of Systematic Reviews (PROSPERO) with registration number CRD420251138714.

### 2.1 Data sources and search strategy

This study systematically searched four major databases: PubMed, EMBASE, Cochrane Library, and Web of Science. The search strategy combined both subject headings and free text terms, covering the following keywords: “Triple Negative Breast Neoplasms”, “Triple Negative Breast Cancer”, “ER-Negative PR-Negative HER2-Negative Breast Cancer”, “ER-Negative PR-Negative HER2-Negative Breast Neoplasms”, “randomized clinical trial”, “immune checkpoint inhibitors”, “Immune Checkpoint Blockers”, “PD-L1 inhibitor”, “PD-1 inhibitor”, “CTLA-4 Inhibitor”, “Cobimetinib”, “Atezolizumab”, “Pembrolizumab”, “Toripalimab”, etc. (**Supplementary Table 2**). The search was conducted from the inception of each database up to August 23, 2025, without language restrictions.

### 2.2 Selection criteria

#### 2.2.1 Inclusion criteria

1. RCTs involving patients with mTNBC diagnosed by histology or cytology;
2. The intervention involved ICIs used alone or in combination as first-line treatment;
3. Comparison of ICIs combined with chemotherapy versus standard treatment (such as chemotherapy alone) for mTNBC in RCTs;
4. The study reports at least one of the following outcomes: overall survival (OS, defined as the time from randomization to death from any cause), progression-free survival (PFS, defined as the time from randomization to disease progression or death from any cause), objective response rate (ORR, defined as the proportion of patients achieving objective response), or ≥ grade 3 adverse events (AE) (based on the National Cancer Institute-common Terminology Criteria for Adverse Events).

#### 2.2.2 Exclusion criteria

1. Duplicate studies published based on the same patient population at different follow-up stages;
2. RCTs that do not explicitly report relevant outcome indicators;
3. Reviews, commentaries, or case reports.

Literature screening was first conducted based on titles and abstracts for preliminary selection, followed by full-text retrieval and evaluation for studies that initially met the eligibility criteria. All included studies were independently cross-verified by two researchers to ensure the accuracy and timeliness of the included data.

### 2.3 Data extraction and quality assessment

Data extraction was independently performed by three researchers following the Preferred Reporting Items for Systematic Reviews and Meta-Analyses (PRISMA) guidelines. In case of any discrepancies, a fourth researcher was consulted to resolve the issues through discussion. From each study, the following information was extracted: trial name, NCT number, journal of publication, randomization ratio, publication year, trial phase, sample size, patient age and racial distribution, and the interventions for each group. The extracted outcomes included the hazard ratios (HR) and 95% confidence intervals (95% CI) for OS and PFS, the odds ratios (OR) and 95% CI for ORR and AE≥3. We used the Revised Cochrane risk-of-bias tool for randomized trials (RoB 2.0) (16) to assess the methodological quality of the included RCTs. This tool is based on five domains: bias arising from the randomization process, bias due to deviations from the intended interventions, bias due to missing outcome data, bias in the measurement of the outcome, and bias in the selection of the reported result. For each study, the risk of bias in each domain was classified as “low risk”, “some concerns” or “high risk”.

### 2.4 Statistical analysis

The primary outcomes of this study are OS and PFS. The secondary outcomes include ORR and AE≥3. In terms of efficacy outcomes, OS and PFS are reported as HR and 95% CI as effect measures. ORR and AE≥3 were expressed as ORs with 95% CIs.

This study employs a Bayesian framework NMA, utilizing the “gemtc (version 4.4)” and “rjags” packages in R software for the analysis. A random-effects model was used to establish four independent Markov Chain Monte Carlo (MCMC) simulation chains, with each chain running 15,000 burn-in iterations to ensure model convergence, followed by 150,000 sampling iterations to obtain posterior distribution estimates. The random-effects model was selected to account for potential between-study heterogeneity across included trials, thereby providing more conservative and robust estimates. To intuitively present the relative efficacy ranking probabilities of different interventions in the NMA, this function calculates the probability of each treatment being in each ranking position (e.g., 1st to Nth place) based on the posterior distribution of the Bayesian network model. The rank.probability() function is used to extract the probability distribution matrix for each treatment across all possible rankings, and a heatmap is drawn using the pheatmap function.

To assess the consistency and inconsistency in the network structure, this study compares the Deviance Information Criterion (DIC) values of the consistency and inconsistency models. A difference in DIC greater than 5 between the two models indicates significant global inconsistency. Additionally, this study performed pairwise meta-analysis using the frequency method in RevMan 5.4 software. Heterogeneity was assessed using the Q test and I² statistic. *I²* ≤ 50% or *P* ≥ 0.1 is considered low heterogeneity, while *I²* > 50% or *P* < 0.1 is considered high heterogeneity. A random-effects model was used for studies with high heterogeneity, while a fixed-effects model was used for studies with low heterogeneity. Sensitivity analysis was conducted for studies with high heterogeneity to confirm the reliability of the results by sequentially excluding individual studies from the model and comparing changes in heterogeneity before and after exclusion. If there was no significant change in heterogeneity, the results were considered reliable. If there was a significant change in heterogeneity, the sources of heterogeneity were analyzed. Funnel plots were used to assess potential publication bias. All statistical tests were two-sided, with a significance level set at α = 0.05.

## 3 Results

### 3.1 Literature screening and characteristics of included studies

**Figure 1:**
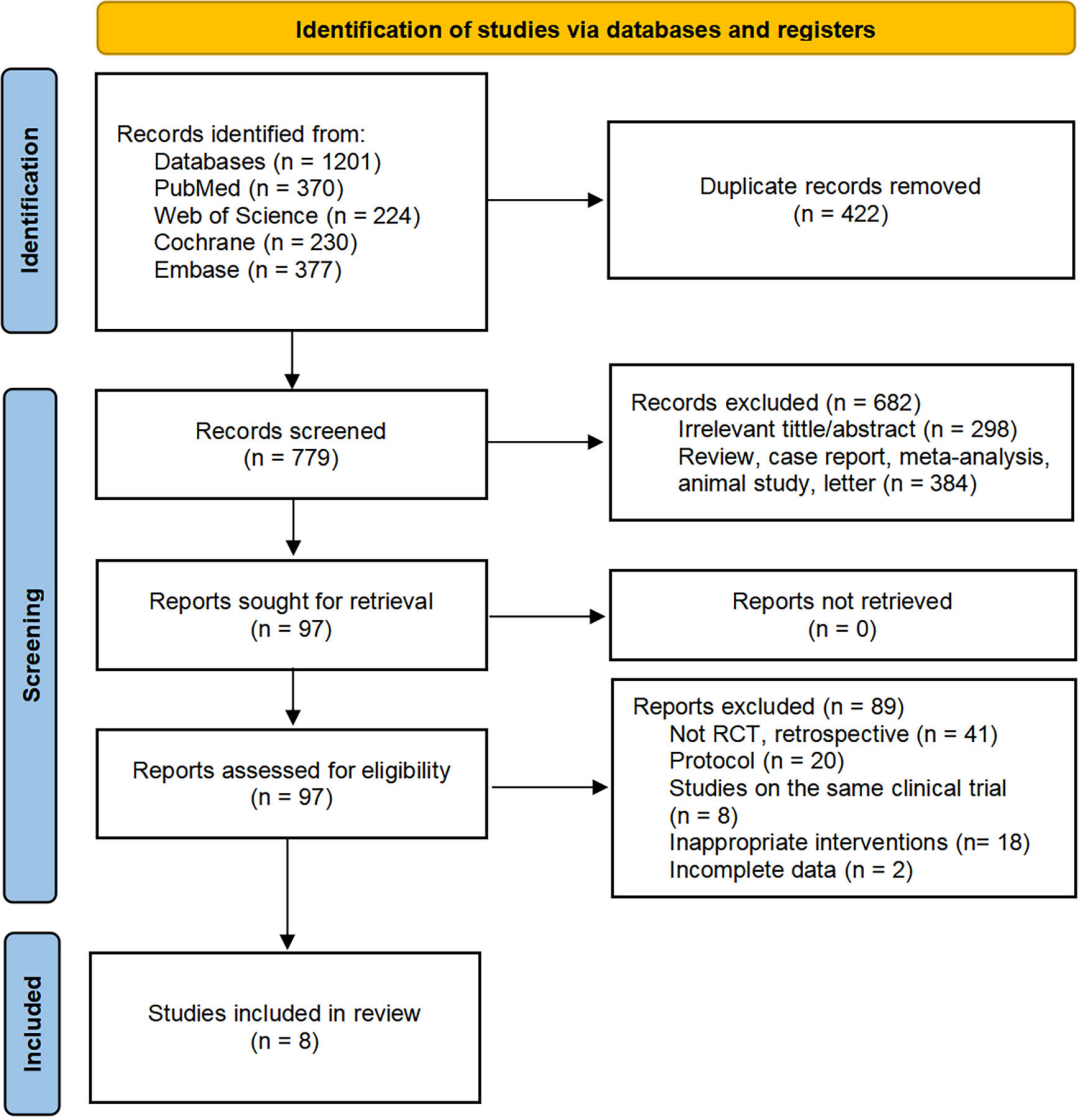
PRISMA flow diagram of study selection.

A total of 1,201 relevant studies were initially identified through the search. After deduplication and abstract screening, irrelevant studies were excluded. Ultimately, 97 studies were deemed eligible for full-text review. Of these, 8 (17–24) studies met the predefined eligibility criteria and were included in the analysis (**Figure 1**). A total of 3789 patients were involved, and the evaluated treatment regimens included 7 types: chemotherapy alone (Chemo), Cobimetinib combined with chemotherapy (Cobime-chemo), Cobimetinib and Atezolizumab combined chemotherapy (Cobime-Atezo-chemo), Atezolizumab combined chemotherapy (Atezo-chemo), Pembrolizumab combined chemotherapy (Pembro-chemo), Atezolizumab and Entinostat combined chemotherapy (Atezo-Entino-chemo), and Toripalimab combined chemotherapy (Toripa-chemo). Detailed characteristics of the included studies are presented in **Tables 1**–**3**.

**Table 1:** Baseline characteristics of studies included in the network meta-analysis.

Study(y)	Source	Registered ID	Sample size	Phase, Design	Randomization	Median Age/y	Ethnicity (n/%)
COLET(2021) (17)	Ann. Oncol	NCT02322814	47/32/31/43	Double-blind,II	1:1:1:1	55/53/52/51	White:(119/77.8%) Asian:(27,17.6%) Other/unknown:(7, 4.6%)
IMpassion130(2021) (18)	Ann. Oncol	NCT02425891	451/451	Double-blind,III	1:1	55/56	White:(609, 67.5%) Asian:(161, 17.8%) Other/unknown:(132, 14.6%)
IMpassion131(2021) (19)	Ann. Oncol	NCT03125902	431/220	Double-blind,III	2:1	54/53	White:(374, 57.5%) Asian:(189, 29.0%) Other/unknown:(88, 13.5%)
KEYNOTE-355(2022) (20)	NEJM	NCT02819518	566/281	Double-blind,III	2:1	NA	NA
ALICE(2022) (21)	Nat. Med	NCT03164993	42/28	Double-blind,IIb	2:3	58.5/52.5	NA
IMpassion132(2024) (22)	Ann. Oncol	NCT03371017	297/298	Double-blind,III	1:1	49/49	White:(281, 73.9%) Asian:(49, 12.9%) Black/African American:(11, 2.9%) American Indian/Alaska Native:(6, 1.6%) Multiple/Unknown:(33,8.7%)
TORCHLIGHT(2024) (23)	ASCO	NCT04085276	353/178	Double-blind,III	2:1	NA	NA
Garrido-Castro(2022) (24)	Cancer Res	NCT02708680	20/20	NA,II	1:1	51/47.5	NA

**Table 2:** Trials and interventions included in the network.

Study(y)	Intervention Arm(s)	Control Arm
COLET(2021) (17)	Arm 1: Cobimetinib 60 mg orally on days 3–23 of each 28-day cycle plus;Paclitaxel 80 mg/m² IV on days 1, 8, and 15 of each 28-day cycle Arm 2: Cobimetinib 60 mg orally on days 3–23 of each 28-day cycle plus; Atezolizumab 840 mg IV on days 1 and 15 of each 28-day cycle plus; Paclitaxel 80 mg/m² IV on days 1, 8, and 15 of each 28-day cycle Arm 3: Cobimetinib 60 mg orally on days 3–23 of each 28-day cycle plus; Atezolizumab 840 mg IV on days 1 and 15 of each 28-day cycle plus; nab-Paclitaxel 100 mg/m² IV on days 1, 8, and 15 of each 28-day cycle	Placebo orally on days 3–23 plus paclitaxel 80 mg/m² IV on days 1, 8, and 15 of each 28-day cycle
IMpassion130(2021) (18)	Atezolizumab 840 mg IV Q2W + nab-Paclitaxel 100 mg/m² IV on days 1, 8, and 15 of a 28-day cycle	Placebo IV Q2W + nab-Paclitaxel 100 mg/m² IV on days 1, 8, and 15 of a 28-day cycle
IMpassion131(2021) (19)	Atezolizumab 840 mg IV Q2W + Paclitaxel 90 mg/m² IV on days 1, 8, and 15 of a 28-day cycle	Placebo IV Q2W + Paclitaxel 90 mg/m² IV on days 1, 8, and 15 of a 28-day cycle
KEYNOTE-355(2022) (20)	Pembrolizumab 200 mg Q3W + Chemotherapy	Placebo Q3W + Chemotherapy
ALICE(2022) (21)	Atezolizumab 840 mg Q2W + Pegylated liposomal doxorubicin (PLD) 20 mg/m² Q2W + Cyclophosphamide 50 mg daily	Placebo Q2W + Pegylated liposomal doxorubicin (PLD) 20 mg/m² Q2W + Cyclophosphamide 50 mg daily
IMpassion132(2024) (22)	Atezolizumab 1200 mg Q3W + chemotherapy (carboplatin + gemcitabine or capecitabine)	Placebo Q3W + chemotherapy (carboplatin + gemcitabine or capecitabine)
TORCHLIGHT(2023) (23)	Toripalimab 240 mg Q3W + nab-Paclitaxel	Placebo Q3W + nab-Paclitaxel
Garrido-Castro(2022) (24)	Atezolizumab 840 mg Q2W + Paclitaxel 80 mg/m² QW	Placebo Q2W + Paclitaxel 80 mg/m² QW

**Table 3:** Characteristics of included randomized controlled trials.

Study	PD-L1≥1% Patients (%)		PD-L1 ≥10% Patients (%)		PD-L1 <1% Patients (%)	
	Intervention(s),n(%)	Control, n (%)	Intervention(s),n(%)	Control, n (%)	Intervention(s),n(%)	Control, n (%)
COLET	7(14.9)/16(50.0)/15(48.4)	11 (25.6)	/	/	26(55.3)/9(28.1)/11(35.5)	18(41.9)
IMpassion130	185(41.0)	184(40.8)	/	/	266(59.0)	267(59.2)
IMpassion131	191(44.3)	101(45.9)	/	/	240(55.7)	119(54.1)
KEYNOTE-355	425(75.1)	211(75.1)	220 (38.9)	103 (36.7)	/	/
ALICE	21(52.5)	10(35.7)	/	/	19(47.5)	17(60.7)
IMpassion132	177(50%)	177(50%)	/	/	/	/

### 3.2 Literature quality assessment

**Figure 2:**
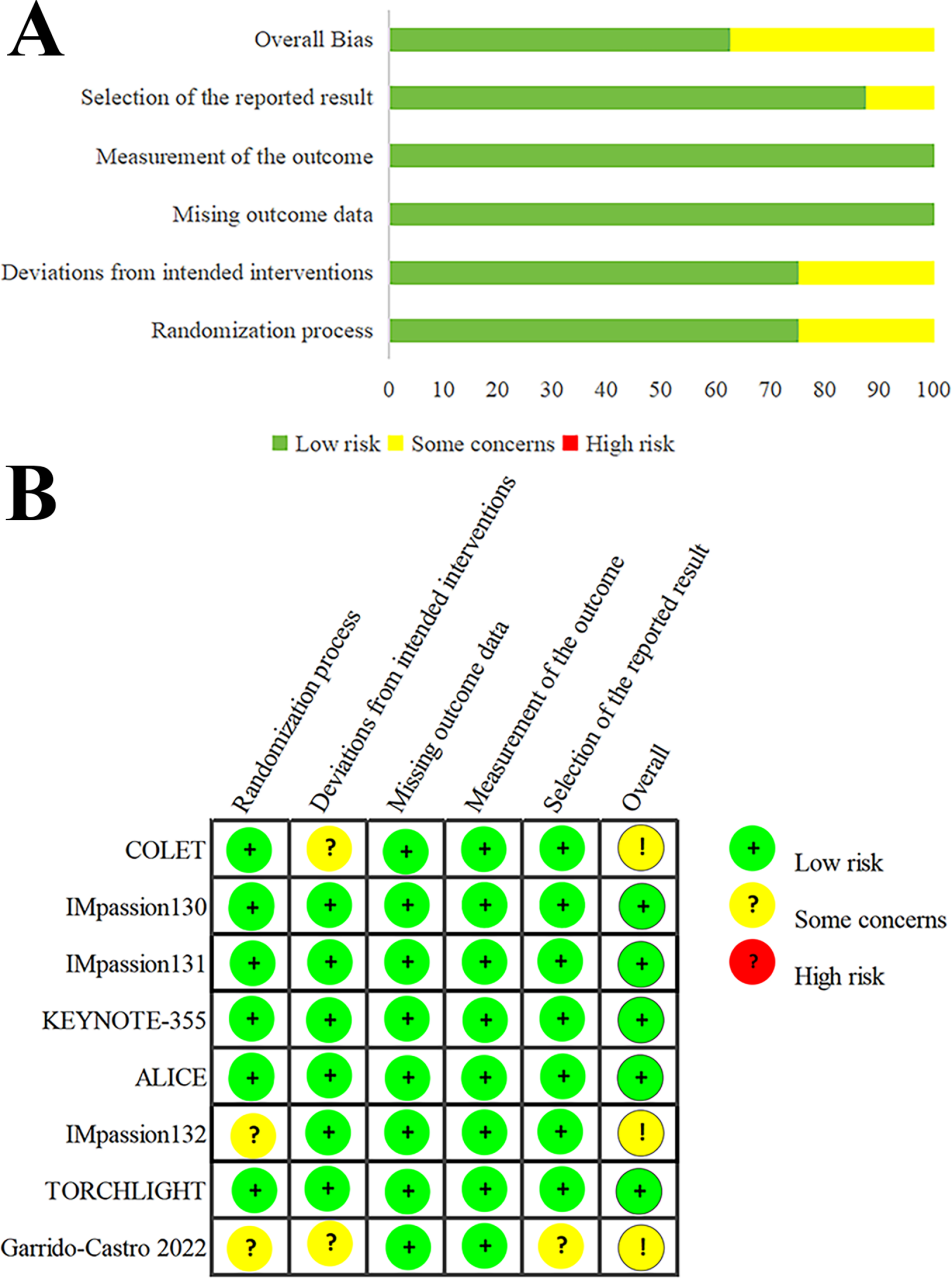
Risk of bias summary and assessments. **(A)** Overview of risk of bias across included trials. **(B)** Domain-specific risk of bias judgments.

The methodological quality of the 8 included studies was evaluated using ROB 2.0. The results showed that 5 studies were assessed as having a “low risk” of bias overall, while 3 studies were rated as having “some concerns.” Specifically, IMpassion132 was rated as “some concerns” due to insufficient description of the random sequence generation and allocation concealment processes. Although COLET mentioned blinding, it did not provide detailed information about the blinding implementation, thus it was also rated as “some concerns”. Garrido-Castro 2022 being a conference abstract, reported only partial prespecified endpoints and did not provide the full study protocol, making it difficult to assess the risk of selective reporting; it was therefore rated as “some concerns.” All studies performed well in terms of intervention adherence and outcome data completeness, with minimal missing data that did not significantly impact the results. Therefore, they were rated as “low risk” in both “deviation from intended interventions” and “missing data” domains. Detailed risk of bias assessments are shown in **Figure 2**.

### 3.3 Pairwise meta-analysis

#### 3.3.1 Comparisons of OS, PFS, ORR, AE≥3

**Figure 3:**
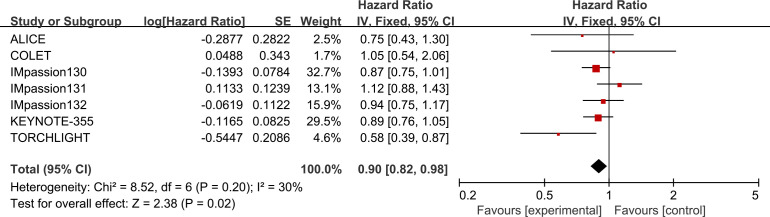
Forest plot of OS comparing ICIs plus chemotherapy versus chemotherapy alone in mTNBC.

Seven studies reported OS, with small statistical heterogeneity between the studies (*P* = 0.2, *I^2^* = 30%), thus a fixed-effect model was used for the meta-analysis. Compared with chemotherapy alone, ICIs combined with chemotherapy significantly improved OS in mTNBC patients (HR = 0.90, 95% CI: 0.82-0.98) (**Figure 3**).

**Figure 4:**
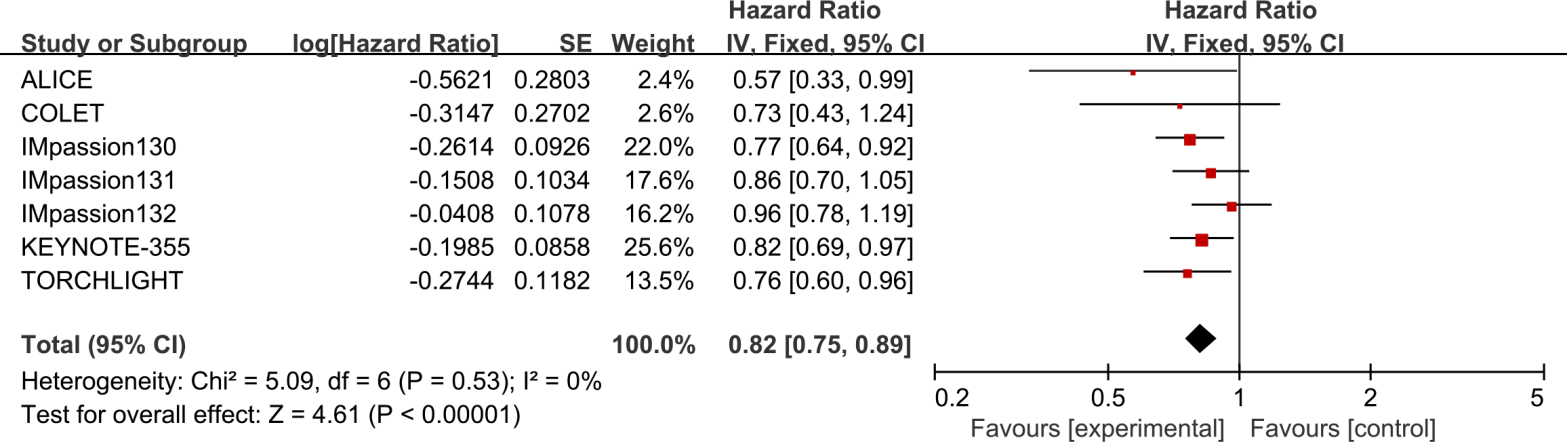
Forest plot of PFS comparing ICIs plus chemotherapy versus chemotherapy alone in mTNBC.

Seven studies reported PFS, with no heterogeneity observed between the studies (*P* = 0.53, *I²* = 0%), so a fixed-effect model was used for the meta-analysis. The results showed that, compared to chemotherapy alone, ICIs combined with chemotherapy significantly improved PFS in mTNBC patients (HR = 0.82, 95% CI: 0.75-0.89) (**Figure 4**).

**Figure 5:**
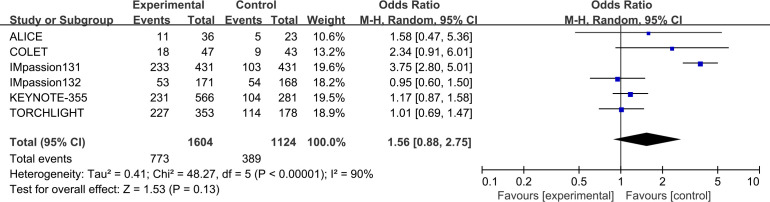
Forest plot of ORR comparing ICIs plus chemotherapy versus chemotherapy alone in mTNBC.

Six studies reported ORR, with significant heterogeneity observed between the studies (*P* < 0.1, *I²* = 90%), thus a random-effects model was used for the meta-analysis. The results showed that although ICIs combined with chemotherapy improved ORR compared to chemotherapy alone (OR = 1.56, 95% CI: 0.88-2.75), the difference did not reach statistical significance (**Figure 5**).

**Figure 6:**
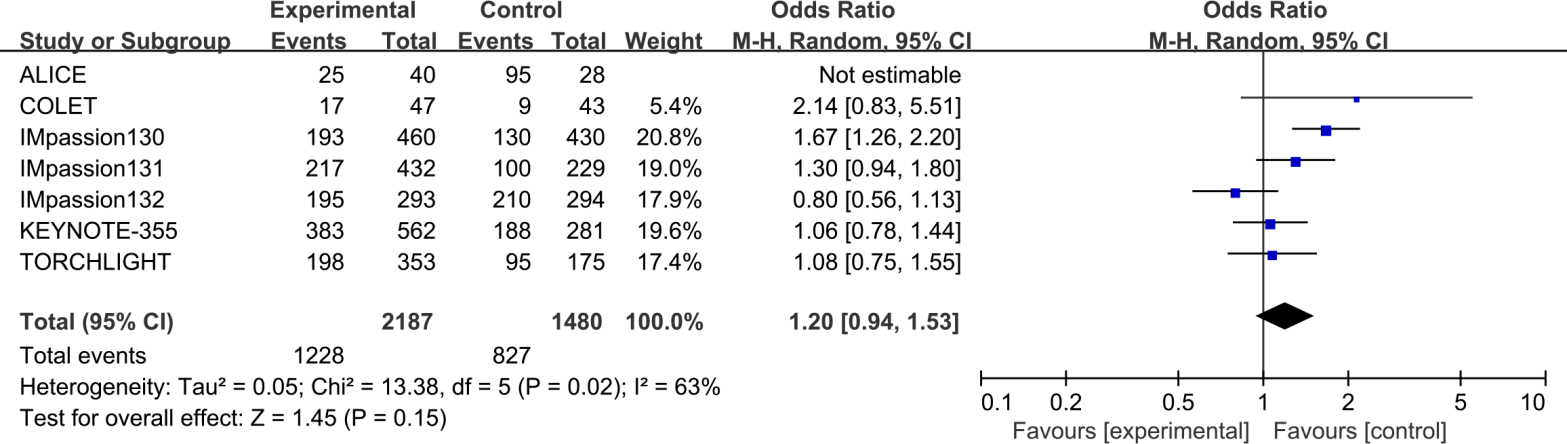
Forest plot of AE ≥3 comparing ICIs plus chemotherapy versus chemotherapy alone in mTNBC.

Seven studies reported AE≥3. There was significant heterogeneity between studies (*P* = 0.02, *I²* = 63%), so a random-effects model was used for the meta-analysis. The results indicated that the incidence of AE≥3 with ICIs combined with chemotherapy was higher than chemotherapy alone (OR = 1.20, 95% CI: 0.94-1.53), although the difference was not statistically significant (**Figure 6**).

#### 3.3.2 Subgroup analysis of PD-L1 expression

**Figure 7:**
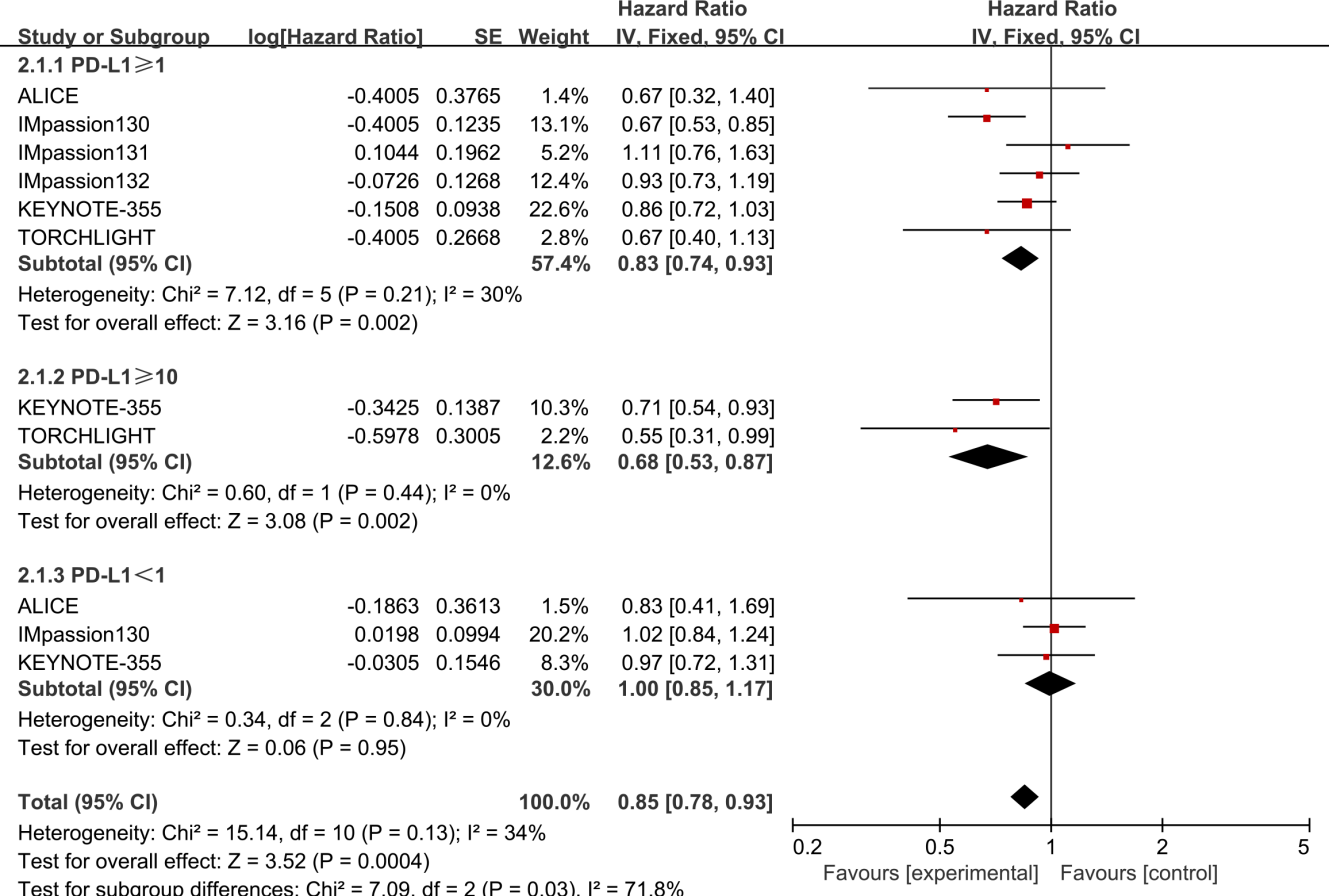
Forest plot of OS by PD-L1 expression subgroup, comparing ICIs plus chemotherapy versus chemotherapy alone in mTNBC.

In patients with PD-L1 positive mTNBC (expression ≥1%), a total of six studies reported OS. There was low heterogeneity observed between the studies (*P* = 0.21, *I²* = 30%), thus a fixed-effects model was used for the meta-analysis. The results showed that compared to chemotherapy alone, ICIs combined with chemotherapy significantly prolonged OS in mTNBC patients (HR = 0.83, 95% CI: 0.74-0.93) (**Figure 7**).

In patients with mTNBC who have high PD-L1 expression (≥10%), two studies reported OS, with no heterogeneity observed between the studies (*P* = 0.44, *I²* = 0%), so a fixed-effects model was used for the meta-analysis. The results showed that, compared to chemotherapy alone, ICIs combined with chemotherapy significantly improved patient OS (HR = 0.68, 95% CI: 0.53-0.87) (**Figure 7**).

In patients with PD-L1 negative (expression < 1%) mTNBC, three studies reported OS, with no heterogeneity observed between the studies (*P* = 0.84, *I²* = 0%), thus a fixed-effects model was used for the meta-analysis. ICIs combined with chemotherapy did not significantly improve OS compared to chemotherapy alone (HR = 1.00, 95% CI: 0.85-1.17), with no statistically significant difference (**Figure 7**).

**Figure 8:**
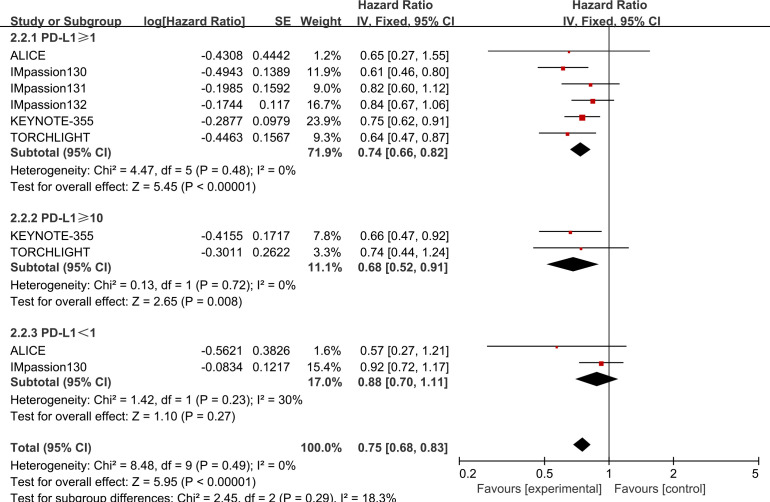
Forest plot of PFS by PD-L1 expression subgroup, comparing ICIs plus chemotherapy versus chemotherapy alone in mTNBC.

In patients with PD-L1 positive mTNBC(expression ≥1%), a total of six studies reported PFS. There was no heterogeneity observed between the studies (*P* = 0.48, *I²* = 0%), so a fixed-effects model was used for the meta-analysis. Compared with chemotherapy alone, ICIs combined with chemotherapy significantly improved PFS in mTNBC patients (HR = 0.74, 95% CI: 0.66-0.82) (**Figure 8**).

In patients with mTNBC who have high PD-L1 expression (≥10%), two studies reported PFS, with no heterogeneity observed between studies (*P* = 0.72, *I²* = 0%). Therefore, a fixed-effect model was used for the meta-analysis. ICIs combined with chemotherapy still significantly prolonged PFS (HR = 0.68, 95% CI: 0.52-0.91) (**Figure 8**).

In patients with PD-L1 negative (expression < 1%) mTNBC, two studies reported PFS, with low heterogeneity between the studies (*P* = 0.23, *I²* = 30%). A fixed-effect model was used for meta-analysis. The results showed that ICIs combined with chemotherapy extended PFS compared to chemotherapy alone (HR = 0.88, 95% CI: 0.70-1.11), although the difference was not statistically significant (**Figure 8**).

### 3.4 Network meta-analyses

**Figure 9:**
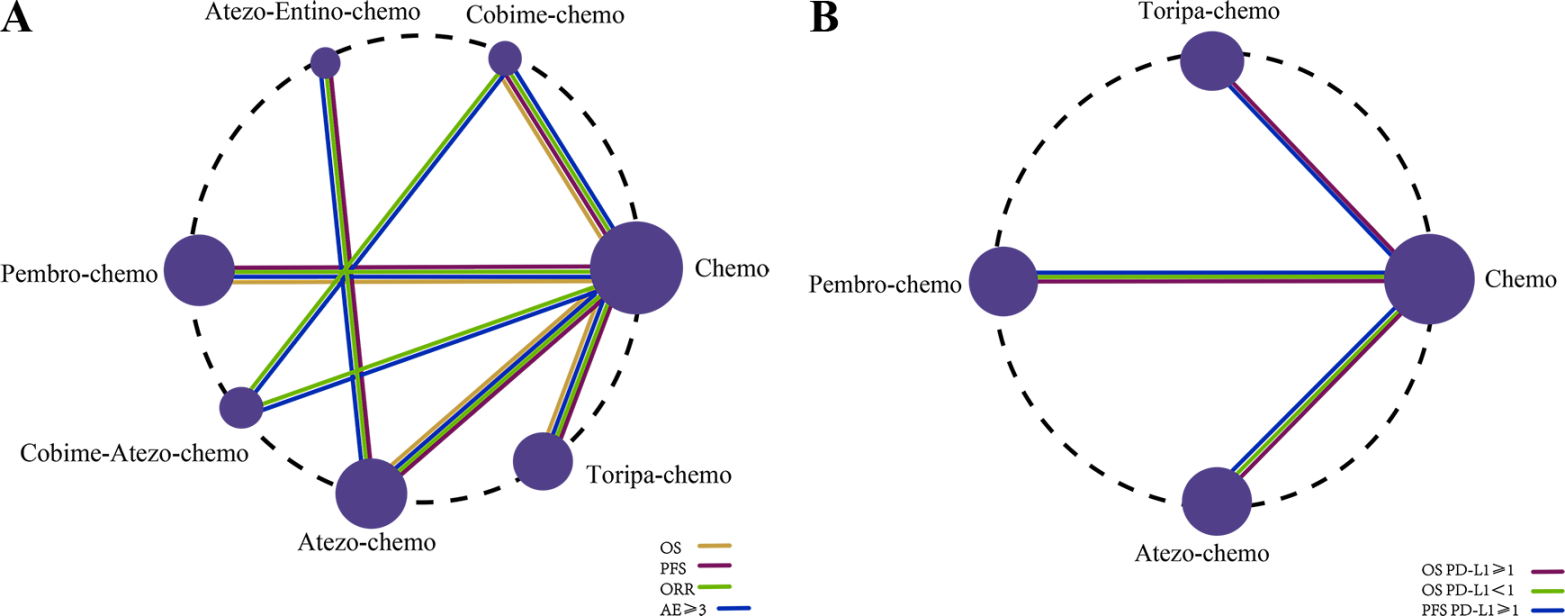
Network plots of treatment comparisons in the network meta-analysis. **(A)** Network of first-line immunotherapy regimens for OS, PFS, ORR, and AE ≥3 in patients with mTNBC. **(B)** Network of first-line immunotherapy regimens stratified by PD-L1 expression.

The primary efficacy outcomes of this study were OS and PFS, while secondary outcomes included ORR and AE≥3. The analysis included comparative networks for four treatment regimens for OS, five for PFS, seven for ORR, and seven for AE≥3 (**Figure 9**).

**Figure 10:**
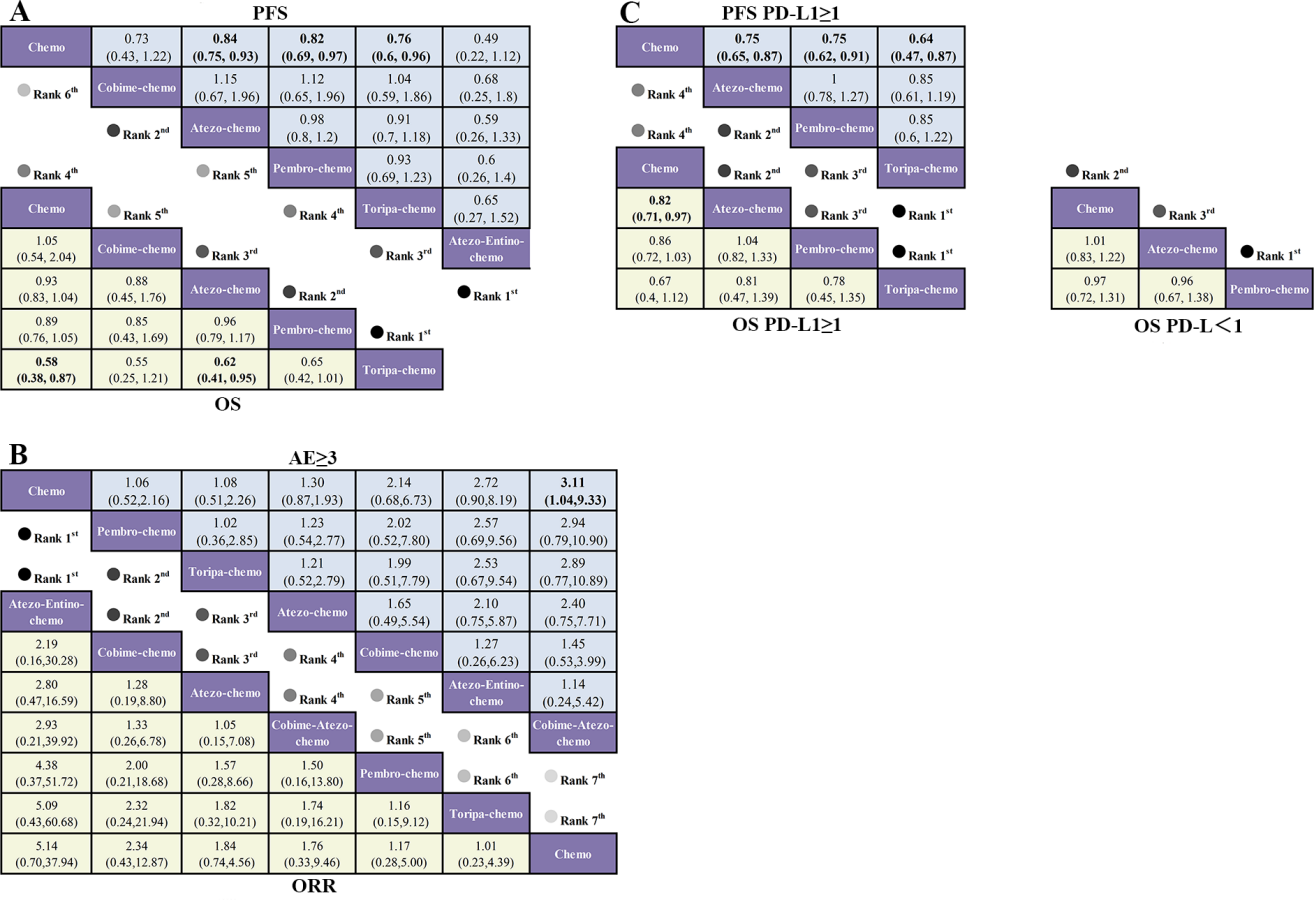
League tables of the comparative efficacy and safety of first-line immunotherapy in patients with mTNBC based on Bayesian network meta-analysis. **(A)** HRs with 95% CIs for OS and PFS. An HR < 1.00 denotes greater survival benefit. **(B)** ORs with 95% CIs for AE ≥3 and ORR. An OR < 1.00 indicates a more favorable outcome. **(C)** HRs with 95% CIs for OS and PFS in patients with PD-L1 expression ≥1% receiving first-line therapy. For patients with PD-L1 <1%, HRs with 95% CIs for OS are shown.

In terms of OS in front-line treatment, only Toripa-chemo showed significant OS benefits compared to chemo (HR = 0.58, 95% CI: 0.38-0.87). Pembro-chemo (HR = 0.89, 95% CI: 0.76-1.05) and Atezo-chemo (HR = 0.93, 95% CI: 0.83-1.04) showed a non-significant improvement in OS, but the differences were not statistically significant. Cobime-chemo did not show any OS improvement compared to chemotherapy (HR = 1.05, 95% CI: 0.54-2.04), though the difference was not statistically significant (**Figure 10A**).

In terms of PFS, compared to chemotherapy, Atezo-chemo (HR = 0.84, 95% CI: 0.75-0.93), Pembro-chemo (HR = 0.82, 95% CI: 0.69-0.97), and Toripa-chemo (HR = 0.76, 95% CI: 0.60-0.96) showed significant PFS benefits. Although Cobime-chemo (HR = 0.73, 95% CI: 0.43-1.22) and Atezo-Entino-chemo (HR = 0.49, 95% CI: 0.22-1.12) demonstrated a trend of improvement, the differences were not statistically significant (**Figure 10A**).

In terms of ORR, compared to chemo, Atezo-Entino-chemo (OR = 5.14, 95% CI: 0.70-37.94), Cobime-chemo (OR = 2.34, 95% CI: 0.43-12.87), and Atezo-chemo (OR = 1.84, 95% CI: 0.74-4.56) all showed an increase in ORR, but the differences were not statistically significant (**Figure 10B**).

In terms of AE≥3, compared to chemotherapy, Pembro-chemo (OR = 1.06, 95% CI: 0.52-2.16) and Toripa-chemo (OR = 1.08, 95% CI: 0.51-2.26) did not significantly increase the incidence of AE≥3. However, Cobime-Atezo-chemo significantly increased the risk of AE≥3 (OR = 3.11, 95% CI: 1.04-9.33) (**Figure 10B**).

In patients with PD-L1 expression ≥1%, only Atezo-chemo showed a significant OS benefit compared to chemotherapy (HR = 0.82, 95% CI: 0.71-0.97). Although Pembro-chemo (HR = 0.86, 95% CI: 0.72-1.03) and Toripa-chemo (HR = 0.67, 95% CI: 0.40-1.12) showed some improvement, the differences were not statistically significant (**Figure 10C**).

In terms of OS in patients with PD-L1 expression < 1%, compared to chemotherapy, Pembro-chemo (HR = 0.97, 95% CI: 0.72-1.31) showed a trend toward improved OS, though the difference was not statistically significant. Atezo-chemo (HR = 1.01, 95% CI: 0.83-1.22) did not demonstrate a significant OS benefit (**Figure 10C**).

In terms of PFS in patients with PD-L1 expression ≥1%, Atezo-chemo (HR = 0.75, 95% CI: 0.65-0.87), Pembro-chemo (HR = 0.75, 95% CI: 0.62-0.91) and Toripa-chemo (HR = 0.64, 95% CI: 0.47-0.87) showed significant improvements in PFS compared to chemotherapy (**Figure 10C**).

### 3.5 Rank

**Figure 11:**
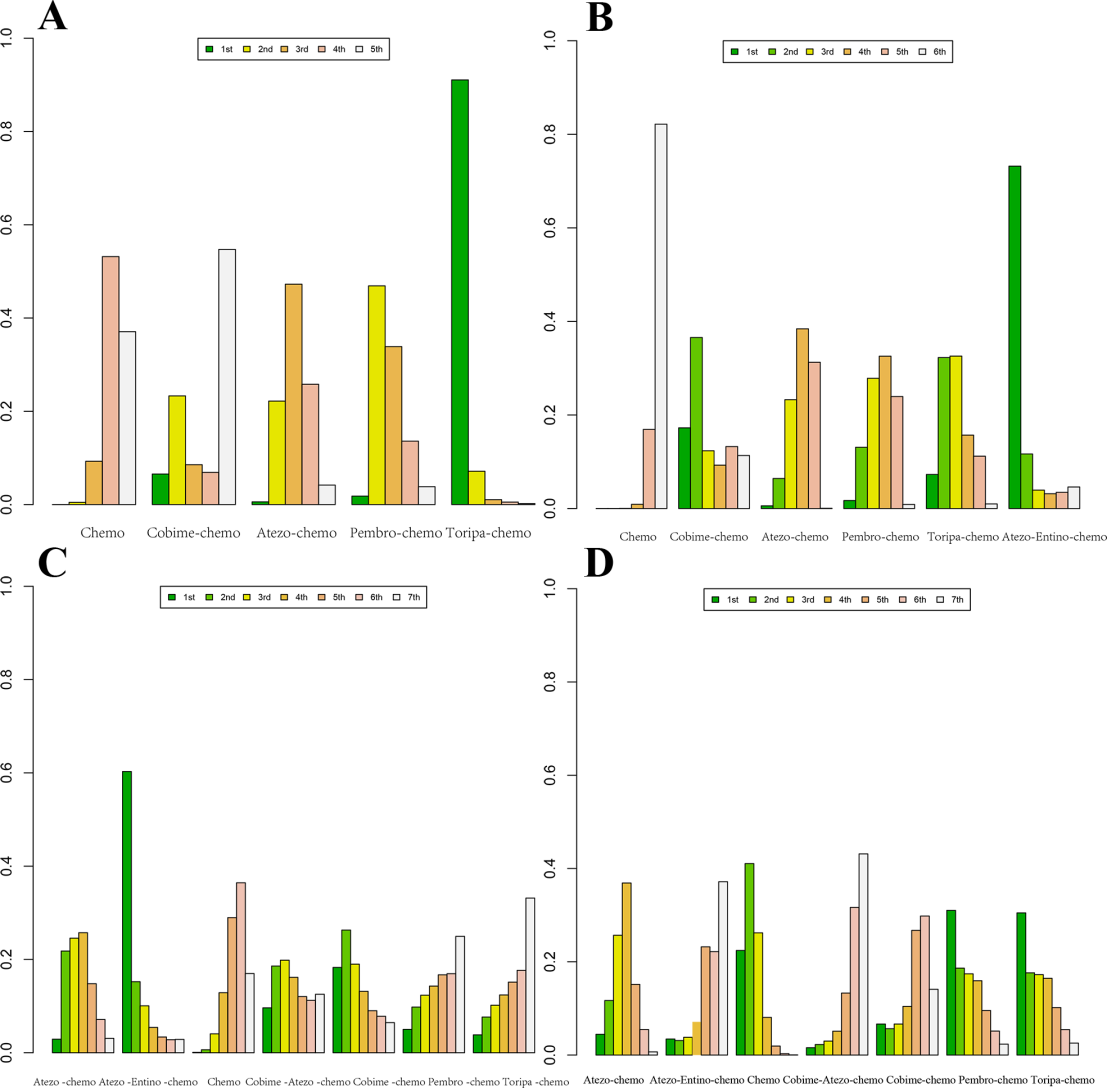
Bayesian ranking profiles for the efficacy and safety of first-line immunotherapy regimens in patients with mTNBC. **(A)** Ranking for OS; **(B)** Ranking for PFS; **(C)** Ranking for ORR; **(D)** Ranking for AE ≥3.

**Figure 12:**
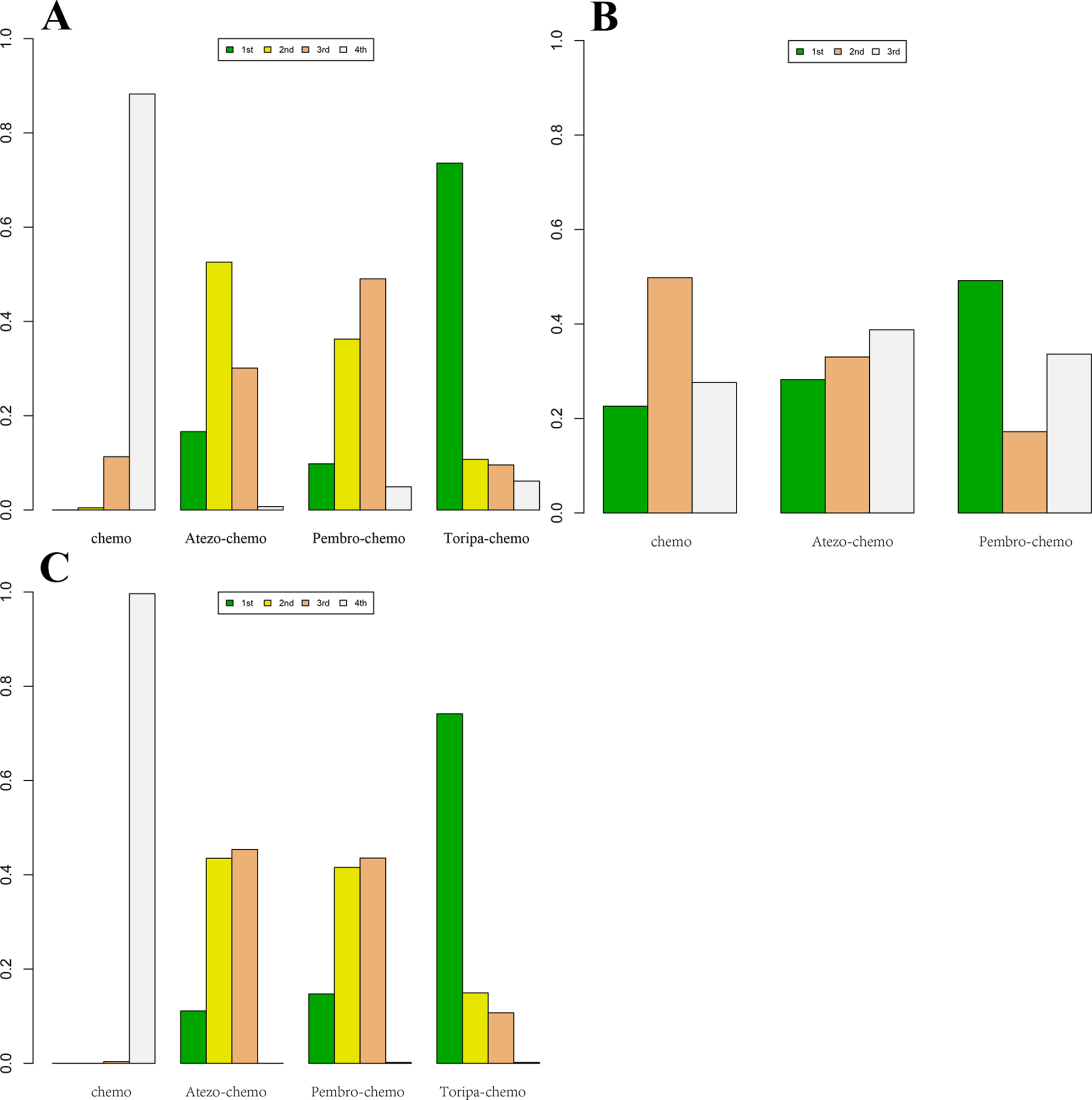
Bayesian ranking profiles for the efficacy and safety of first-line immunotherapy regimens in patients with mTNBC, stratified by PD-L1 expression. **(A)** OS ranking in patients with PD-L1 ≥1%; **(B)** OS ranking in patients with PD-L1 <1%; **(C)** PFS ranking in patients with PD-L1 ≥1%.

Based on the Bayesian ranking probability analysis results (**Figures 11**, **12**; **Supplementary Figures 1**-**7**), for first-line treatment of mTNBC patients, Toripa-chemo had the highest probability of ranking first in OS (91.07%). Atezo-Entino-chemo ranked first in PFS and ORR with probabilities of 73.19% and 60.29%. Pembro-chemo had the best safety ranking for AE≥3, with a probability of 31.01%, while Toripa-chemo ranked second(30.48%). In patients with PD-L1 expression ≥1%, Toripa-chemo had the highest probability of ranking first in OS (73.59%) and PFS (74.16%). In patients with PD-L1 expression <1%, Pembro-chemo had the highest probability of being the optimal regimen for OS (49.19%). The ranking results should be interpreted in conjunction with effect estimates rather than in isolation.

### 3.6 Convergence, consistency, sensitivity, and publication bias

The Gelman-Rubin statistic and diagnostic plots were used to assess model convergence. The potential scale reduction factor (PSRF) values for all parameters were close to 1.00 (generally <1.05), indicating satisfactory convergence of the Markov Chain Monte Carlo (MCMC) simulations. In addition, trace plots showed good mixing and stability across chains, and density plots demonstrated consistent posterior distributions, further supporting the reliability of the model. (**Supplementary Figures 8**-**21**). To verify the consistency of the network structure, this study compared the consistency and inconsistency models using the DIC. As shown in **Table 4**, the DIC differences for all outcomes were less than 5, indicating no significant inconsistency was detected. The exchangeability between direct and indirect comparison results was deemed acceptable. Notably, for the OS outcome in the PD-L1 ≥1% subgroup, a moderate difference in DIC values was observed between the consistency and inconsistency models (12.33 vs. 10.28). However, this difference remained below the commonly accepted threshold of 5, suggesting that the inconsistency was not substantial. Considering both statistical criteria and the clinical plausibility of the network structure, the consistency model was therefore adopted as the primary analytical model. Nonetheless, the observed difference may indicate a certain degree of local heterogeneity or sparse evidence within this subgroup, and the results should be interpreted with caution.

**Table 4:** Comparison of DIC values between consistency and inconsistency models across different clinical endpoints in patients with mTNBC.

Endpoints	Model type	Dbar	pD	DIC	I2
OS	Consistency	7.58	4.01	11.59	21%
	Inconsistency	6.66	5.65	12.31	10%
PFS	Consistency	9.38	5.00	14.38	25%
	Inconsistency	8.20	6.79	14.99	15%
ORR	Consistency	14.98	14.84	29.81	7%
	Inconsistency	14.75	14.43	29.18	5%
AE≥3	Consistency	17.33	16.61	33.94	8%
	Inconsistency	16.92	16.39	33.31	5%
OS PD-L1≥1	Consistency	9.33	3.00	12.33	46%
	Inconsistency	6.10	4.18	10.28	18%
OS PD-L1<1	Consistency	2.32	2.02	4.34	14%
	Inconsistency	2.40	2.16	4.56	16%
PFS PD-L1≥1	Consistency	6.54	2.97	9.52	24%
	Inconsistency	5.35	4.55	9.90	7%

DIC, deviance information criterion; pD, effective number of parameters; I², heterogeneity statistic.

In traditional pairwise meta-analysis, sensitivity analysis was performed by sequentially excluding individual studies. The results showed that the combined effect size did not change significantly, indicating that the results are robust. Funnel plots for OS, PFS, ORR, and AE≥3 were generated for assessment, and their shapes were largely symmetrical with no apparent outliers, suggesting a low likelihood of small sample bias or publication bias (**Supplementary Figures 22**-**27**).

## 4 Discussion

### 4.1 Principal findings

This study primarily aimed to identify the optimal first-line immunotherapy strategies for mTNBC, particularly across different PD-L1 expression subgroups. This study provides a comprehensive evaluation of systematic reviews and network meta-analysis assessing the efficacy and safety of first-line immunotherapy combination chemotherapy regimens for mTNBC, with a comprehensive evaluation of benefit differences across various PD-L1 expression levels (≥1%, ≥10%, and <1%). Our findings provide the following clinical insights:

1. Compared with chemotherapy, ICIs combined with chemotherapy provide superior survival benefits for mTNBC patients, with no significant difference in safety. Patients with high PD-L1 expression benefit more significantly: for OS, the HR for populations with PD-L1≥1% and ≥10% were 0.83 and 0.68, respectively, while the overall population is 0.90. For PFS, the HR for the corresponding populations were 0.74 and 0.68, with the overall population at 0.82, indicating that the therapeutic benefit of immunotherapy increases with higher PD-L1 expression levels.
2. In first-line treatment of mTNBC, ICIs combined with chemotherapy significantly improve OS and PFS compared to chemotherapy alone, with an increasing trend in ORR, although it did not reach statistical significance. The incidence of AE≥3 was slightly higher, but the difference was not statistically significant.
3. Toripa-chemo and Pembro-chemo show a balanced performance between efficacy and safety, making them relatively ideal treatment options. Notably, Toripa-chemo demonstrated clear survival benefit in PD-L1 positive patients. Cobime-chemo also showed promising efficacy potential and acceptable safety, making it an alternative treatment option. In patients with PD-L1≥1%, Toripa-chemo showed best performance in OS and PFS. In contrast, in patients with PD-L1<1%, Pembro-chemo showed the most favorable trend in OS benefit.

In the first-line treatment of mTNBC, ICIs combined with chemotherapy have shown significant improvements in OS, PFS, and ORR. The potential mechanisms may involve chemotherapy agents, such as taxanes, inducing immunogenic cell death (ICD), which releases tumor-associated antigens and activates dendritic cells and T cells, thereby enhancing the anti-tumor immune response (25, 26). ICIs block the PD-1/PD-L1 pathway, relieving T cell inhibition and restoring their anti-tumor activity (27). Additionally, certain chemotherapy drugs, such as gemcitabine, can reduce myeloid-derived suppressor cells (MDSCs) and regulatory T cells (Tregs), restoring the ratio of effector to regulatory T cells (Teff/Treg) and improving the tumor microenvironment. While some of (25) this evidence comes from other tumor types, they still provide a reasonable explanation for the synergistic effects of combination therapy (28). On the other hand, although the incidence of AE≥3 is slightly increased in combination therapy, the difference is not statistically significant. According to the expert consensus of the Society for Immunotherapy of Cancer (SITC), most immune-related adverse events (irAEs) are mild to moderate, and early identification and intervention are typically effective in managing them. These events are generally reversible and manageable, indicating that the overall safety of combination therapy remains acceptable (29, 30). These findings further support the synergistic role of chemotherapy and immunotherapy in improving clinical outcomes.

Toripa-chemo demonstrates superior survival benefits compared to other ICIs, which may be attributed to its molecular characteristics, such as a higher PD-1 binding affinity, sustained receptor occupancy, and enhanced T-cell activation potential (31). Toripalimab, a humanized anti-PD-1 monoclonal antibody, specifically blocks the interaction between PD-1 and PD-L1/PD-L2, reversing T-cell dysfunction and boosting the anti-tumor immune response (32, 33). Additionally, its survival advantage may also be influenced by the study design and population characteristics. The key supporting study, TORCHLIGHT, primarily involved an Asian (Chinese) population, whereas studies like KEYNOTE-355 and IMpassion130 included a broader global population. There may be racial differences in the molecular subtype distribution, tumor mutation burden, and immune microenvironment of TNBC, which could impact the efficacy of different ICIs. On the other hand, the combination of Toripalimab with albumin-bound paclitaxel may also enhance efficacy, as paclitaxel is believed to have better immune-modulatory activity, such as promoting antigen presentation and reducing Treg cell proportion, potentially contributing to a synergistic effect with immunotherapy (34).

In the first-line treatment of mTNBC, patients with high PD-L1 expression benefit significantly from ICIs combined with chemotherapy, as demonstrated by prolonged OS and PFS. When PD-L1 is highly expressed on tumor cell surfaces, it can inhibit T cell immune activity by binding to PD-1 on T cells, promoting tumor immune escape (35). Furthermore, high PD-L1 expression is often associated with higher levels of tumor-infiltrating lymphocytes (TILs) in the tumor microenvironment (36). This immune environment is more conducive to the action of ICIs, further supporting the role of PD-L1 as a biomarker for predicting the efficacy of ICIs (37).

Although Cobime-Atezo-chemo can significantly improve OS and PFS, it also significantly increases the risk of AE≥3. ICIs restore T cell function and activate the immune system to attack tumors by blocking immunosuppressive signals such as PD-1/PD-L1 (35, 38). However, this activated immune response lacks specificity and may also attack normal tissues, leading to immune-related adverse events (irAEs), such as colitis, pneumonia, hepatitis, and endocrine diseases (39). These irAEs can reach grade 3 or higher, and their mechanism (autoimmune attack) is fundamentally different from the non-specific cytotoxic reactions of traditional chemotherapy (such as bone marrow suppression and gastrointestinal symptoms) (40, 41). Furthermore, dual immune blockade strategies (such as simultaneous inhibition of CTLA-4 and PD-1) may further enhance immune activation, while simultaneously increasing the risk and severity of irAEs by providing a more comprehensive release of immune suppression (42).

Compared with Zhang et al. and several previous Meta-analyses (43–45), this study draws similar conclusions in terms of PFS benefits and safety, but there are differences in OS. This study shows that immunotherapy combined with chemotherapy can significantly improve OS (HR = 0.90, 95% CI: 0.82-0.98), while Zhang et al. did not observe a significant difference (HR = 0.90, 95% CI: 0.78-1.04). This difference may stem from the fact that the previous analyses included fewer OS data from the studies, which could not establish a significant OS benefit, whereas this study included more recent and comprehensive clinical trials (such as TORCHLIGHT, IMpassion132, etc.), providing more mature and extensive survival data. Additionally, by employing a Bayesian network meta-analysis approach, we were able to more thoroughly assess the efficacy and safety of various immunotherapy regimens. Similarly, Yang et al.’s (46) meta-analysis confirmed that in first-line treatment of mTNBC, PD-1/PD-L1 inhibitors combined with chemotherapy can significantly improve OS and PFS compared to chemotherapy alone, with manageable safety. However, this study expands on existing evidence in several ways. First, we included more recently published RCTs with larger sample sizes and systematically evaluated subgroups with different PD-L1 expression levels. Second, by using Bayesian network meta-analysis, we not only reconfirm the survival benefits of immunotherapy combination therapy but also for the first time to comprehensively compare the efficacy differences among different immune checkpoint inhibitors through probabilistic ranking, finding that Toripalimab combined with chemotherapy performs best in terms of overall survival.

### 4.2 Implications

This study is the first to use Bayesian network meta-analysis to systematically compare the efficacy and safety of different immunotherapy combination chemotherapy regimens in first-line treatment of mTNBC, with a particular focus on comprehensive assessments of subgroups with different PD-L1 expression levels (including ≥1%, ≥10%, and <1%). The included literature primarily consists of high-quality, large-scale RCTs, showing a low overall risk of bias that ensures the reliability and transparency of the results. As ICIs have been recommended by various international guidelines as standard first-line treatment for eligible mTNBC patients, and with the continuous publication of the latest clinical trial data for new ICIs like Toripalimab and their combination regimens, clinicians require updated evidence to guide treatment decisions. This study conducted the first indirect comparisons and probability ranking of multiple ICI regimens, including Pembrolizumab, Atezolizumab, and Toripalimab, which not only validated the recommendations of existing guidelines but also provided timely evidence to support the application of new drugs and combination strategies. The study results indicate that in the PD-L1 positive population, Toripalimab combined with chemotherapy shows potential to become the optimal treatment regimen in terms of OS and PFS, providing a basis for future updates to treatment guidelines. Furthermore, by analyzing the efficacy across populations with different PD-L1 threshold expressions, this study further clarifies that patients with high PD-L1 expression can derive significant survival benefits from immunotherapy combinations, reinforcing the stratification value of PD-L1 as a biomarker in personalized treatment, which aids in precision therapy and maximizes patient benefits in clinical practice. Finally, by integrating both efficacy and safety outcomes, we find that the safety of most immunotherapy regimens is generally manageable. However, certain treatment strategies, such as Cobimetinib combined with Atezolizumab and chemotherapy, are significantly associated with an increased risk of high-grade adverse events. This finding provides key insights for clinical decision-making, helping to balance efficacy and toxicity, and supporting the development of more personalized treatment strategies that optimize both efficacy and quality of life for patients.

### 4.3 Limitations

Despite striving for comprehensiveness and rigor, there are still several limitations. First, a key limitation is the heterogeneity of chemotherapy backbones across the included trials. Different regimens were combined with different cytotoxic agents, such as nab-paclitaxel, paclitaxel, or gemcitabine plus carboplatin, and these chemotherapy partners may differ in their intrinsic antitumor activity as well as in their immunomodulatory effects. Therefore, the observed differences between treatment regimens in this network meta-analysis may not be attributable solely to the immune checkpoint inhibitors themselves, but may also have been influenced by the efficacy of the accompanying chemotherapy backbone. Although all comparisons were made against trial-specific control arms and prior evidence suggests that in the treatment of advanced BC, the survival benefit of albumin-bound paclitaxel compared to standard paclitaxel may not be statistically significant, suggesting that the differences in chemotherapy regimens may have a limited impact on overall outcomes (47), this source of heterogeneity cannot be fully eliminated in an indirect comparison framework. Accordingly, the comparative results should be interpreted with caution, especially when making cross-trial inferences regarding the relative efficacy of different immunotherapy regimens. Secondly, the racial distribution of the patients included in this study is imbalanced, with a predominance of Caucasian and Asian populations, and underrepresentation of minority groups such as Black and Hispanic populations. Therefore, the generalizability of the conclusions to these underrepresented groups requires further validation. Future studies should include more diverse populations from different racial backgrounds to provide more universally applicable evidence. Thirdly, although we systematically searched and included all relevant RCTs on first-line immunotherapy for mTNBC, some interventions (such as Toripa-chemo, Pembro-chemo, etc.) are still based on data from single studies. While these trials are mostly well-designed, large-sample Phase III studies with relatively complete baseline data reporting, a certain degree of uncertainty remains in the results, which may impact the robustness of related comparisons. Another important limitation is the heterogeneity in PD-L1 testing assays and positivity definitions across the included trials. Different studies used different PD-L1 platforms, such as SP142 and 22C3, and these assays are not fully interchangeable. SP142 mainly evaluates PD-L1 expression on immune cells, whereas 22C3 commonly relies on the combined positive score (CPS). Consequently, the PD-L1-positive subgroups defined in different trials may not be completely equivalent, which may reduce the comparability of indirect subgroup analyses in this network meta-analysis. Therefore, the subgroup findings based on PD-L1 status should be interpreted cautiously, particularly when informing cross-trial clinical decision-making. Despite these limitations, the present study remains focused on providing a comparative framework to identify optimal first-line immunotherapy strategies in mTNBC. In addition, this study did not include emerging treatment strategies involving antibody–drug conjugates (ADCs) combined with immunotherapy, such as the ASCENT-04 trial evaluating sacituzumab govitecan plus immune checkpoint inhibitors. These combination regimens have recently shown promising efficacy and are becoming part of the standard-of-care for PD-L1-positive metastatic TNBC in certain regions. Therefore, the present analysis may not fully reflect the latest therapeutic landscape, and future studies incorporating ADC-based combinations are warranted.

## Data Availability

The original contributions presented in the study are included in the article/**Supplementary Material**. Further inquiries can be directed to the corresponding author.
